# Predicting ineffective thrombolysis in acute ischemic stroke with clinical and biochemical markers

**DOI:** 10.1038/s41598-024-64413-w

**Published:** 2024-06-11

**Authors:** Yinglei Li, Ning Li, Yuanyuan Zhou, Litao Li

**Affiliations:** 1 Department of Neurology, Hebei Medical University, Shijiazhuang, China; 2 Department of Neurology, Affiliated Hospital of Hebei University, Baoding, China; 3 Department of Emergency Medicine, Baoding No.1 Central Hospital, Baoding, China; 4 Department of Neurology, Baoding No.1 Central Hospital, Baoding, China; 5 Department of Neurology, Hebei General Hospital, Shijiazhuang, China; 6 Hebei Provincial Key Laboratory of Cerebral Networks and Cognitive Disorders, Hebei General Hospital, Shijiazhuang, China

**Keywords:** Ischemic stroke, Thrombolysis, recombinant tissue plasminogen activator (rtPA), Predictive model, LASSO regression, Personalized medicine, Medical research, Neurology

## Abstract

**Ischemic stroke remains a leading cause of morbidity and mortality globally. Despite the advances in thrombolytic therapy, notably recombinant tissue plasminogen activator (rtPA), patient outcomes are highly variable. This study aims to introduce a novel predictive model, the Acute Stroke Thrombolysis Non-Responder Prediction Model (ASTN-RPM), to identify patients unlikely to benefit from rtPA within the critical early recovery window. We conducted a retrospective cohort study at Baoding No.1 Central Hospital including 709 adult patients diagnosed with acute ischemic stroke and treated with intravenous alteplase within the therapeutic time window. The ASTN-RPM was developed using Least Absolute Shrinkage and Selection Operator (LASSO) regression technique, incorporating a wide range of biomarkers and clinical parameters. Model performance was evaluated using Receiver Operating Characteristic (ROC) curves, calibration plots, and Decision Curve Analysis (DCA). ASTN-RPM effectively identified patients at high risk of poor response to thrombolysis, with an AUC of 0.909 in the training set and 0.872 in the validation set, indicating high sensitivity and specificity. Key predictors included posterior circulation stroke, high admission NIHSS scores, extended door to needle time, and certain laboratory parameters like homocysteine levels. The ASTN-RPM stands as a potential tool for refining clinical decision-making in ischemic stroke management. By anticipating thrombolytic non-response, clinicians can personalize treatment strategies, possibly improving patient outcomes and reducing the burden of ineffective interventions. Future studies are needed for external validation and to explore the incorporation of emerging biomarkers and imaging data.

## Introduction

Ischemic stroke remains a formidable challenge to global health, being a leading cause of long-term disability and mortality worldwide^1–4^. The complexity of stroke pathophysiology necessitates early and effective interventions to mitigate its devastating outcomes^5,6^.

In this context, thrombolytic therapy, specifically the administration of tissue plasminogen activator (rtPA)^7–9^, has revolutionized the acute management of ischemic stroke, significantly improving outcomes when administered within a narrow therapeutic window. Despite this advancement, the clinical efficacy of thrombolytic treatment varies substantially among individuals, with a significant proportion of patients not achieving optimal recovery.

This variability highlights the urgent need for predictive models that can accurately identify patients unlikely to benefit from thrombolysis, enabling the personalization of treatment approaches to avoid ineffective interventions and guide alternative therapeutic strategies. Recent research efforts have focused on identifying predictors of long-term outcomes post-thrombolysis^10–14^, exploring a range of biomarkers and clinical parameters. However, the predictive value of short-term neurological recovery, particularly within the first week following treatment, has not been sufficiently addressed. Early recovery is a critical determinant of overall prognosis and can guide clinicians in making informed decisions regarding subsequent care and rehabilitation strategies.

Moreover, the integration of comprehensive clinical data into predictive models remains a largely untapped resource, potentially limiting the utility of these models in real-world clinical settings.

To bridge these gaps, our study introduces the “Acute Stroke Thrombolysis Non-Responder Prediction Model” (ASTN-RPM), utilizing the Least Absolute Shrinkage and Selection Operator (LASSO) regression technique. This advanced statistical method enables the selection of the most prognostically significant variables from a vast array of potential predictors, focusing specifically on those that can accurately forecast the short-term outcomes of thrombolytic therapy. ASTN-RPM specifically aims to predict neurological function recovery within seven days post-treatment, a critical yet underexplored period in stroke recovery literature.

Through rigorous variable selection, ASTN-RPM has been developed as an innovative predictive model, with its efficacy and clinical relevance thoroughly evaluated using an array of methodological tools, including nomograms, Receiver Operating Characteristic (ROC) curves, calibration plots, and Decision Curve Analysis (DCA). This comprehensive evaluation provides a holistic assessment of its predictive accuracy and practical utility in clinical decision-making.

By focusing on the critical need for early prognostic indicators and leveraging the full potential of available clinical data, our study not only contributes to the existing body of knowledge on stroke management but also introduces ASTN-RPM as a practical tool for clinicians. This model is designed to refine therapeutic decision-making processes, with the ultimate goal of improving patient outcomes, reducing the burden of unnecessary medical interventions, and significantly enhancing the quality of life for those affected by ischemic stroke.

## Materials and methods

### Study design and participants

This retrospective cohort study was conducted at Baoding No.1 Central Hospital and involved the collection of patient data from January 2020 to October 2023, with the aim of developing and validating a predictive model to identify acute ischemic stroke patients who are less likely to respond to intravenous alteplase therapy. Non-responsiveness was determined by an unchanged or increased National Institutes of Health Stroke Scale (NIHSS) score from baseline to day 7 post-treatment, indicating a lack of improvement or worsening of neurological function.

Eligible participants were adults aged 18 and over, diagnosed with acute ischemic stroke, and administered intravenous alteplase within 3 to 4.5 h of symptom onset. Exclusions were made for patients who underwent endovascular treatments after alteplase, did not exhibit definitive focal hyperintensities on diffusion-weighted imaging, had incomplete medical records, had hospital stays under 7 days, or presented with malignancies, autoimmune diseases, significant organ failure, or active infections at stroke onset.

The study protocol was reviewed and approved by the Institutional Review Board (IRB) of Baoding No.1 Central Hospital. Given the study's retrospective nature, involving the analysis of existing patient records, the IRB granted a waiver for the requirement of informed consent. This decision was based on the ethical principle that the waiver would not adversely affect the rights and welfare of the participants, as the study involved no more than minimal risk. All patient data was de-identified and handled confidentially to ensure privacy and compliance with ethical standards. We confirm that all research methods were carried out in accordance with the guidelines and regulations pertinent to retrospective cohort studies and ethical oversight as outlined by the Declaration of Helsinki and local legislation. The waiver does not affect any of the patients' medical care, as the research only involved the collection and analysis of pre-existing data.

### Data collection

Data collection for this investigation involved a detailed retrospective analysis of acute ischemic stroke patients treated with intravenous alteplase at Baoding No.1 Central Hospital, spanning from January 2020 to October 2023. Post-treatment, patients were systematically divided into two subsets for model development and validation—namely, a training set and a validation set—through a process of random assignment. This approach ensured an equitable distribution of essential demographic and clinical attributes across both groups.

The dataset encompassed a broad range of variables critical for model construction, including the NIHSS scores to quantify neurological impairment, alongside demographic details (age and gender) and lifestyle factors (smoking and drinking status). Medical history variables incorporated previous stroke occurrences, diabetes mellitus, atrial fibrillation, coronary artery disease, hyperlipidemia, and hypertension. Baseline physiological measurements taken into account were systolic and diastolic blood pressures and body mass index (BMI).

An extensive laboratory profile was compiled for each patient, detailing complete blood counts (including white blood cell, hemoglobin, and platelet counts), neutrophil and monocyte counts, and the relevant calculated ratios such as the platelet-to-neutrophil ratio and neutrophil-to-lymphocyte ratio. Metabolic and biochemical parameters—specifically, fasting glucose levels, renal function (creatinine), uric acid, lipid profile (total cholesterol, triglycerides, LDL, and HDL cholesterol levels), and homocysteine levels—were also thoroughly documented. The detailed TOAST classification system was employed to categorize stroke etiology, and information was also gathered on the presence of posterior circulation stroke, highlighting the comprehensive and multifaceted nature of the dataset used to underpin the predictive model’s development.

### Statistical methodology

In the development of the Acute Stroke Thrombolysis Non-Responder Prediction Model (ASTN-RPM), our statistical analysis rigorously evaluated predictors of thrombolytic therapy outcomes in acute ischemic stroke patients. A cohort of 709 patients was randomly divided into two groups for model development and validation: 497 individuals in the training dataset and 212 in the validation dataset, maintaining a roughly 7:3 ratio. This division ensured a balanced data distribution for comprehensive model training and validation.

For continuous variables, the normality of the data distribution was assessed using the Shapiro–Wilk test. Based on the results, normally distributed continuous variables were analyzed using the Student's t-test, while non-normally distributed continuous variables were analyzed using the Mann–Whitney U test, ensuring sensitive analysis of the underlying data structure. Categorical variables were examined using the χ2 test or Fisher’s exact test, as appropriate, to accurately portray demographic and clinical characteristics across outcome groups.

The core of our analysis utilized LASSO logistic regression for its efficiency in selecting variables within a high-dimensional dataset and its ability to reduce model complexity and overfitting by penalizing the magnitude of regression coefficients. This technique helped identify a subset of variables most predictive of thrombolytic therapy ineffectiveness in acute ischemic stroke patients. The optimal penalty parameter (lambda) was determined through ten-fold cross-validation aimed at minimizing prediction error. Variables deemed significant via LASSO regression were further analyzed using a multivariable logistic regression model to determine their independent associations with therapy ineffectiveness.

To construct the nomogram, we used the significant predictors identified from the multivariable logistic regression model. The nomogram was developed using the 'rms' package in R, which allowed us to visualize the predictive model and quantify the impact of each predictor variable on the likelihood of thrombolytic therapy ineffectiveness. Each variable's contribution was determined by its regression coefficient, which represents the relative weight or importance of that variable in predicting the outcome. The nomogram assigns points to each predictor based on its coefficient, and the total points correspond to a predicted probability of thrombolysis ineffectiveness.

The predictive performance of ASTN-RPM was assessed through Receiver Operating Characteristic (ROC) curve analysis, with the Area Under the Curve (AUC) as a critical measure of its discriminatory ability. Model calibration was evaluated using calibration plots to confirm the consistency between observed outcomes and predictions. Additionally, Decision Curve Analysis (DCA) measured the clinical usefulness of ASTN-RPM across various decision thresholds. All statistical analyses were conducted using R software (version 4.3.0), utilizing specific packages such as 'glmnet' for LASSO regression, 'pROC' for ROC curve analysis, and 'rms' for calibration plots, thereby enhancing the reproducibility of our findings. Significance was determined at a p-value of less than 0.05.

Through this comprehensive statistical approach, the ASTN-RPM was thoroughly developed, aiming to significantly improve clinical decision-making and patient management in the acute stroke setting.

## Result

### Baseline characteristics

**Figure 1:**
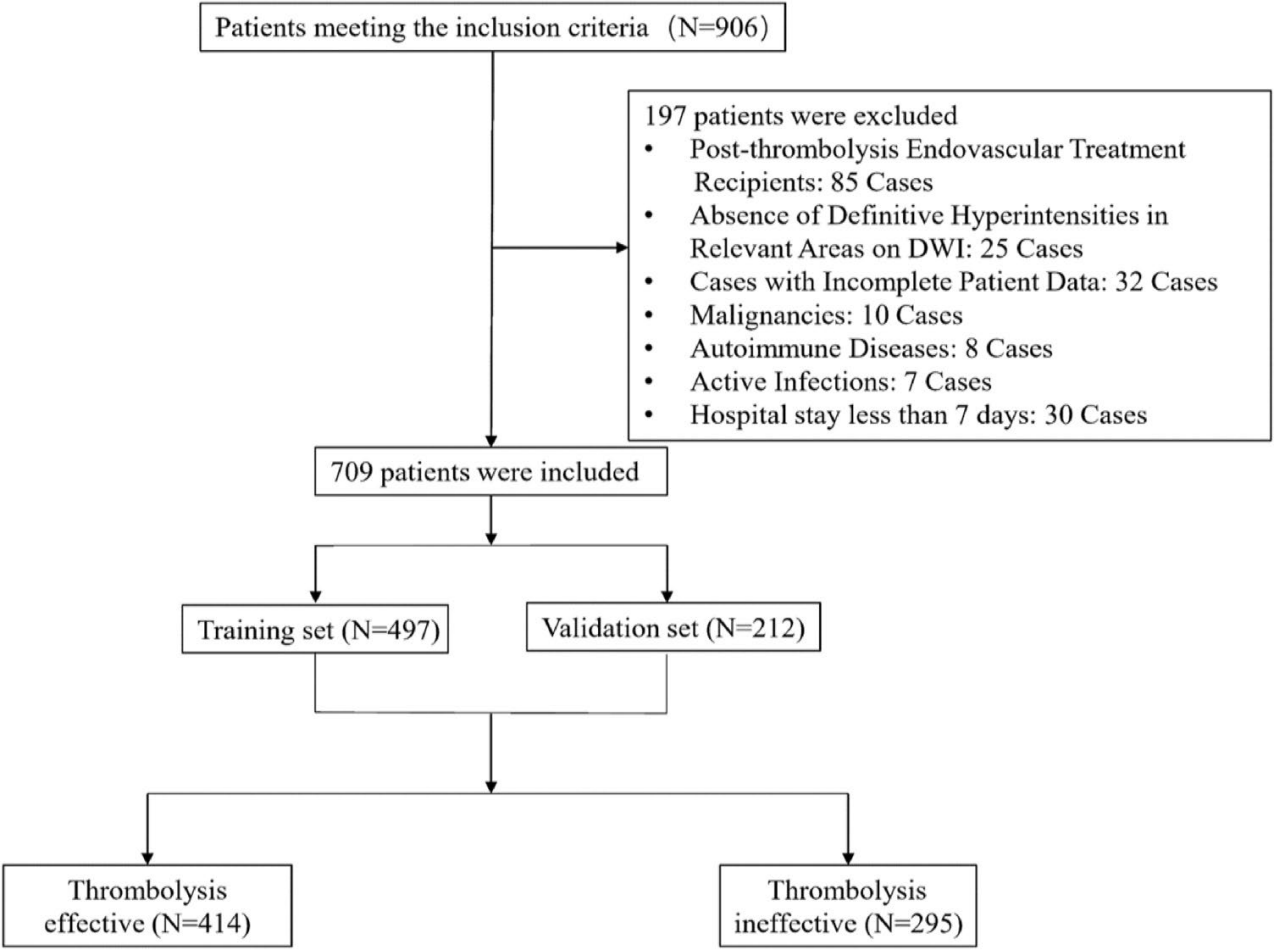
Enrollment and Allocation of Study Participants. Flowchart depicting the enrollment of 906 patients, exclusions applied, and the final inclusion of 709 patients into the training (n = 497) and validation (n = 212) sets for the predictive model development.

Between January 2020 and October 2023, our study initially involved 906 participants eligible for inclusion. Post-application of exclusion criteria, the cohort was narrowed to 709 patients for analysis, as illustrated in Fig. 1. For the purposes of our analysis, patients were categorized into “effective” (n = 414) and “ineffective” (n = 295) thrombolysis groups based on predefined criteria assessing the therapeutic response to intravenous alteplase (Table1). Table 1 outlines the demographic and clinical attributes at baseline for both cohorts. Additionally, the patients were divided into a training set (n = 497) and a validation set (n = 212), maintaining a roughly 7:3 ratio. There were no significant differences between the two groups in terms of various baseline characteristics (Supplemental Table 1).

**Table 1:** Baseline Characteristics of Patients Undergoing Thrombolysis.

Variables	Total (n = 709)	Thrombolysis effective (n = 414)	Thrombolysis ineffective (n = 295)	p
TOAST classification of stroke etiology n (%)				< 0.001
Large artery atherosclerosis	536 (76)	298 (72)	238 (81)	
Small vessel disease	108 (15)	95 (23)	13 (4)	
Cardioembolic	55 (8)	14 (3)	41 (14)	
Other determined etiology	5 (1)	5 (1)	0 (0)	
Undetermined etiology	5 (1)	2 (0)	3 (1)	
Posterior circulation stroke, n (%)				0.015
NO	550 (78)	335 (81)	215 (73)	
YES	159 (22)	79 (19)	80 (27)	
Smoking status, n (%)				< 0.001
NO	368 (52)	244 (59)	124 (42)	
YES	341 (48)	170 (41)	171 (58)	
Drinking status, n (%)				0.006
NO	480 (68)	263 (64)	217 (74)	
YES	229 (32)	151 (36)	78 (26)	
History of hypertension, n (%)				0.999
NO	268 (38)	157 (38)	111 (38)	
YES	441 (62)	257 (62)	184 (62)	
Gender, n (%)				0.43
Female	237 (33)	133 (32)	104 (35)	
Male	472 (67)	281 (68)	191 (65)	
Admission impaired consciousness, n (%)				< 0.001
NO	574 (81)	389 (94)	185 (63)	
YES	135 (19)	25 (6)	110 (37)	
Prior stroke history, n (%)				0.99
NO	619 (87)	362 (87)	257 (87)	
YES	90 (13)	52 (13)	38 (13)	
Diabetes mellitus, n (%)				< 0.001
NO	547 (77)	339 (82)	208 (71)	
YES	162 (23)	75 (18)	87 (29)	
Atrial fibrillation history, n (%)				< 0.001
NO	623 (88)	384 (93)	239 (81)	
YES	86 (12)	30 (7)	56 (19)	
Coronary artery disease history, n (%)				0.002
NO	552 (78)	340 (82)	212 (72)	
YES	157 (22)	74 (18)	83 (28)	
History of hyperlipidemia, n (%)				0.705
NO	702 (99)	409 (99)	293 (99)	
YES	7 (1)	5 (1)	2 (1)	
Admission NIHSS, Median (Q1,Q3)	3 (1, 9)	2 (1, 3)	9 (4, 13.5)	< 0.001
Age (years), Median (Q1,Q3)	65 (55, 71)	64 (55, 70)	66 (56, 73)	0.007
Height (cm), Median (Q1,Q3)	170 (162, 173)	170 (162, 173)	170 (162, 173)	0.878
Weight (Kg), Median (Q1,Q3)	70 (60, 78)	70 (63, 78)	70 (60, 78)	0.469
Body Mass Index, Median (Q1,Q3)	25.06 (22.49, 27.17)	25.18 (22.6, 27.06)	24.65 (22.16, 27.4)	0.388
Door to Needle Time (min), Median (Q1,Q3)	49 (35, 73)	45 (33, 62.75)	60 (37, 90)	< 0.001
Systolic blood pressure (mmHg), Median (Q1,Q3)	146 (134, 160)	143 (133, 156)	150 (136, 161)	< 0.001
Diastolic blood pressure (mmHg), Median (Q1,Q3)	82 (76, 89)	82 (76, 89)	82 (76, 89)	0.995
White blood cell count (× 10^9^/L), Median (Q1,Q3)	7.4 (6.01, 8.93)	7.31 (6.01, 8.74)	7.57 (5.99, 9.21)	0.228
Hemoglobin (g/L), Mean ± SD	144.19 ± 17.41	146.19 ± 16.31	141.37 ± 18.51	< 0.001
Platelet count (× 10^9^/L), Median (Q1,Q3)	216 (179, 256)	219 (186, 257)	212 (172, 250.5)	0.061
Neutrophil count (× 10^9^/L), Median (Q1,Q3)	4.49 (3.44, 5.84)	4.43 (3.45, 5.56)	4.73 (3.42, 6.44)	0.021
Monocyte count (× 10^9^/L), Median (Q1,Q3)	0.52 (0.4, 0.64)	0.49 (0.38, 0.6)	0.56 (0.43, 0.72)	< 0.001
Platelet to Neutrophil Ratio, Median (Q1,Q3)	47.55 (35.61, 65.31)	49.85 (38.73, 66.86)	43.9 (31.66, 62.74)	0.003
Neutrophil percentage, Median (Q1,Q3)	62.9 (53.8, 72.8)	61.55 (53.23, 71)	65.8 (55.5, 75.3)	0.003
Lymphocyte percentage, Median (Q1,Q3)	1.87 (1.36, 2.5)	1.96 (1.43, 2.59)	1.74 (1.3, 2.4)	0.009
Neutrophil to Lymphocyte Ratio, Median (Q1,Q3)	2.25 (1.5, 3.82)	2.1 (1.45, 3.48)	2.59 (1.57, 4.38)	0.002
Systemic Immune Inflammation Index, Median (Q1,Q3)	493.14 (321.7, 819.31)	466.78 (313.82, 763.42)	549.67 (329.43, 904.52)	0.019
Prothrombin Time (s), Median (Q1,Q3)	10.8 (10.3, 11.5)	10.7 (10.1, 11.4)	11 (10.35, 11.6)	0.001
Prothrombin Activity (%), Median (Q1,Q3)	100.7 (93.9, 108.7)	102 (95.9, 108.7)	100.3 (91.8, 108.4)	0.003
Activated Partial Thromboplastin Time (s), Median (Q1,Q3)	26.4 (24.6, 29)	26.4 (24.6, 28.58)	26.5 (24.6, 29.3)	0.338
Fasting Blood Glucose (mmol/L), Median (Q1,Q3)	6.86 (5.81, 8.97)	6.63 (5.69, 8.33)	7.29 (6.04, 9.79)	< 0.001
Brain natriuretic peptide (pg/ml), Median (Q1,Q3)	28.3 (9.8, 114)	23 (7.9, 60.8)	56 (12.75, 166)	< 0.001
Lactate Dehydrogenase (U/L), Median (Q1,Q3)	399 (233.9, 490.61)	385.92 (207.02, 469.49)	422.06 (301.48, 513.62)	< 0.001
Creatinine (μmol/L), Median (Q1,Q3)	67.1 (57.44, 78.73)	65.93 (57.23, 78.07)	68.6 (57.55, 79.32)	0.284
Uric Acid (μmol/L), Median (Q1,Q3)	323.58 (264.9, 395.47)	330.04 (272.62, 398.4)	318.2 (254.6, 393.92)	0.109
Total Cholesterol (mmol/L), Median (Q1,Q3)	4.56 (3.92, 5.3)	4.55 (3.92, 5.22)	4.57 (3.93, 5.36)	0.706
Triglycerides (mmol/L), Median (Q1,Q3)	1.28 (0.92, 1.8)	1.33 (0.95, 1.83)	1.24 (0.88, 1.72)	0.04
Low-Density Lipoprotein (mmol/L), Median (Q1,Q3)	2.7 (2.12, 3.28)	2.67 (2.12, 3.24)	2.73 (2.1, 3.36)	0.615
HDL/LDL ratio, Median (Q1,Q3)	0.37 (0.29, 0.48)	0.41 (0.33, 0.5)	0.32 (0.22, 0.43)	< 0.001
Homocysteine (μmol/L), Median (Q1,Q3)	14.86 (11.5, 25.2)	12.9 (10.5, 16.65)	22.96 (14.58, 27.43)	< 0.001
High Density Lipoprotein (mmol/L), Median (Q1,Q3)	1.02 (0.79, 1.23)	1.09 (0.94, 1.28)	0.82 (0.63, 1.1)	< 0.001
Total Cholesterol/HDL Cholesterol ratio, Median (Q1,Q3)	4.47 (3.65, 5.5)	4.17 (3.54, 4.9)	5.09 (4.03, 7.62)	< 0.001

This table summarizes demographic, clinical, and laboratory characteristics of 709 patients, categorized into thrombolysis effective (n = 414) and ineffective (n = 295) groups. Variables include TOAST classification, stroke specifics, lifestyle factors, medical history, admission details, and initial laboratory findings. Differences were assessed using appropriate statistical tests.

This study evaluated 45 potential predictors associated with the outcomes of intravenous thrombolysis in acute ischemic stroke. Significant disparities were observed across several variables, suggesting their potential impact on thrombolytic outcomes. Notably, the TOAST classification revealed marked differences, with large artery atherosclerosis, small vessel disease, and cardioembolic strokes exhibiting distinct distributions between the effective and ineffective thrombolysis groups (p < 0.001), indicating the stroke's etiology as a critical factor in therapeutic response. The incidence of posterior circulation stroke also differed significantly, suggesting an anatomical influence on treatment efficacy (p = 0.015).

Lifestyle factors showed significant variations, particularly with smoking being more prevalent in the thrombolysis ineffective group (58%) compared to the effective group (41%). This difference highlights the potential impact of smoking status on the outcomes of thrombolysis, underscoring its importance as a predictive factor in treatment effectiveness (p < 0.001). Comorbid conditions such as diabetes mellitus and atrial fibrillation were significantly more common in the ineffective group, underscoring the influence of underlying health conditions on the success of thrombolytic therapy (p < 0.001).

The presence of impaired consciousness at admission was notably higher in the ineffective group (37% vs. 6%), pointing to the severity of neurological impairment as a determinant of thrombolytic effectiveness (p < 0.001). Furthermore, laboratory parameters, including hemoglobin levels and neutrophil counts, alongside admission NIHSS scores, presented significant differences between groups, reinforcing their importance in predicting thrombolysis outcomes. The median NIHSS score at admission notably highlighted the divergence in initial neurological status between the effective and ineffective groups, with higher scores associated with ineffective thrombolysis (p < 0.001).

Conversely, variables such as gender, history of hypertension, hyperlipidemia, and certain physiological measurements (height, weight, body mass index) showed no significant differences between groups, indicating a lesser or negligible impact on the efficacy of thrombolysis in this cohort.

### Variable selection

**Figure 2:**
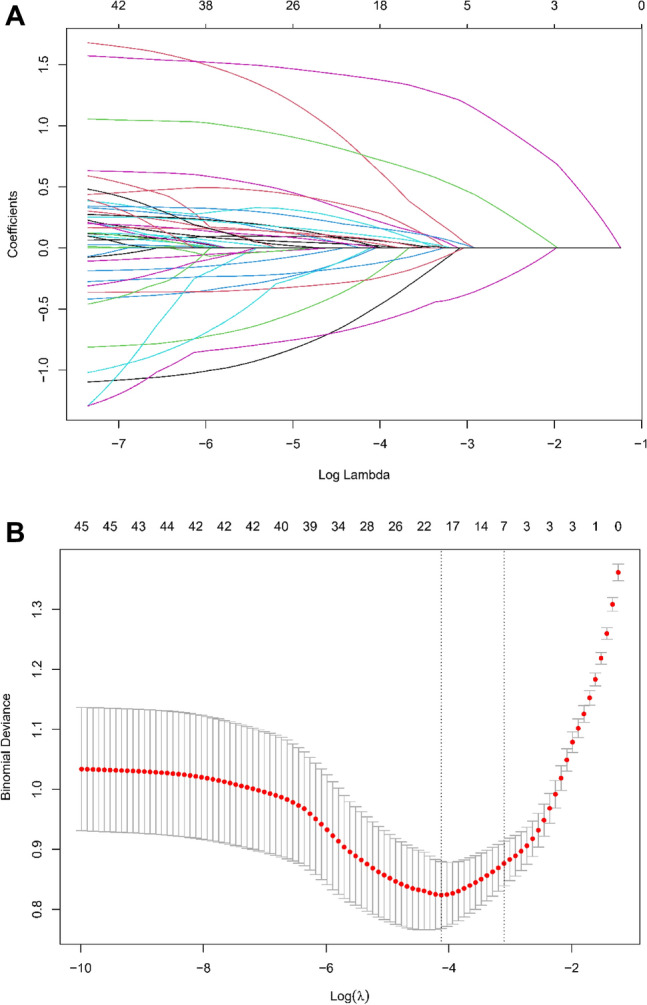
(**A**) LASSO Regression Coefficient Profiles. LASSO regression coefficient profiles of the variables against the log lambda sequence. Each line represents a variable with its coefficient shrinking towards zero as lambda increases. The optimal lambda value is chosen based on cross-validation, beyond which the coefficients are penalized towards zero and excluded from the model. (**B**) Cross-Validation for Lambda Selection in LASSO Regression. Plot of the cross-validated binomial deviance against log(lambda) values in the training set for LASSO regression. The red dots represent the average deviance for each lambda, and the vertical dotted lines define the optimal value of lambda that minimizes the deviance (one standard error rule). This optimal lambda is used for variable selection in the model.

In our extensive study aimed at discerning the variables that significantly influence the outcomes of intravenous thrombolysis, we thoroughly evaluated a dataset comprising 45 variables. This dataset spanned demographic, clinical history, and comprehensive laboratory measurements. To refine the selection of variables and mitigate the risk of overfitting, we employed the LASSO regression technique using the glmnet package in R, coupled with ten-fold cross-validation to determine the optimal regularization parameter (λ). The selection of λ adhered to the one standard error rule, applied to minimize cross-validation error, thereby ensuring a balance between the model's simplicity and its predictive accuracy (Fig. 2A,B).

Through this analytical rigor, we distilled the variables to a concise set that shows a statistically significant correlation with thrombolysis outcomes. These pivotal variables include Posterior circulation stroke, Smoking status, Admission impaired consciousness, Admission NIHSS, Hemoglobin, Homocysteine, Door to Needle Time, High Density Lipoprotein, and the HDL/LDL ratio (Table 2). Each of these retained variables, exhibiting non-zero coefficients in our LASSO model, plays a critical role in elucidating the complex interplay between systemic biological processes and the effectiveness of intravenous thrombolysis.

**Table 2:** Variable Coefficients from LASSO Regression for ASTN-RPM.

Variable	Variable coefficient
Intercept	0.407
Posterior circulation stroke	0.020
Smoking status	0.0224
Admission impaired consciousness	0.009
Admission NIHSS	0.210
Hemoglobin	− 0.008
Homocysteine	0.084
Door to Needle Time	0.009
High Density Lipoprotein	− 0.075
HDL/LDL ratio	0.0027

This table presents coefficients for variables predictive of thrombolysis outcome from LASSO logistic regression. Includes clinical and laboratory predictors such as posterior circulation stroke, smoking status, and admission NIHSS score among others.

### Multivariable analysis

In our detailed multivariable logistic regression analysis, aimed at uncovering factors significantly influencing the effectiveness of thrombolytic therapy, we further investigated variables initially pinpointed through LASSO regression. Our refined analysis illuminated several predictors significantly associated with an increased likelihood of thrombolysis ineffectiveness. These included Posterior circulation stroke, Smoking status, higher Admission NIHSS scores, elevated Homocysteine levels, extended Door to Needle Time, and lower levels of High-Density Lipoprotein (HDL).

These findings shed light on the crucial demographic, clinical, and biological determinants that interplay to influence the outcomes of intravenous thrombolysis. Notably, our analysis underscored the significance of Posterior circulation stroke, Smoking status, and particularly, higher Admission NIHSS and elevated Homocysteine levels as prominent factors increasing the risk of ineffective thrombolysis. Furthermore, longer Door to Needle Times were also identified as critical, signifying the importance of timely treatment initiation in enhancing thrombolytic therapy's success. Conversely, lower HDL levels were associated with adverse thrombolysis outcomes, highlighting a potential avenue for pre-treatment assessment and intervention. The Odds Ratios (ORs), Confidence Intervals (CIs), and p-values elucidating these relationships are comprehensively detailed in Table 3. Through this multifaceted analysis, we elucidate the complexities underlying thrombolytic therapy outcomes, offering insights into optimizing patient evaluation and treatment protocols in acute stroke management.

**Table 3:** Multivariable Logistic Regression for Thrombolysis Outcome Prediction.

	B	SE	OR	CI	Z	*P*
Posterior circulation stroke	0.408	0.203	1.57	1.10–2.82	2.173	0.011
Smoking status	0.672	0.272	1.96	1.15–3.34	2.470	0.014
Admission NIHSS	1.69	0.22	5.42	3.52–8.34	7.671	< 0.001
Homocysteine	0.894	0.175	2.44	1.73–3.44	5.094	< 0.001
Door to Needle Time	0.354	0.13	1.42	1.1–1.84	2.728	0.006
High Density Lipoprotein	-0.498	0.222	0.61	0.39–0.94	− 2.248	0.025

Details results from multivariable logistic regression analysis identifying independent predictors of thrombolytic therapy success. Shows regression coefficient, standard error, odds ratio with confidence intervals, and p-values for significant predictors.

### Development of a predictive nomogram

**Figure 3:**
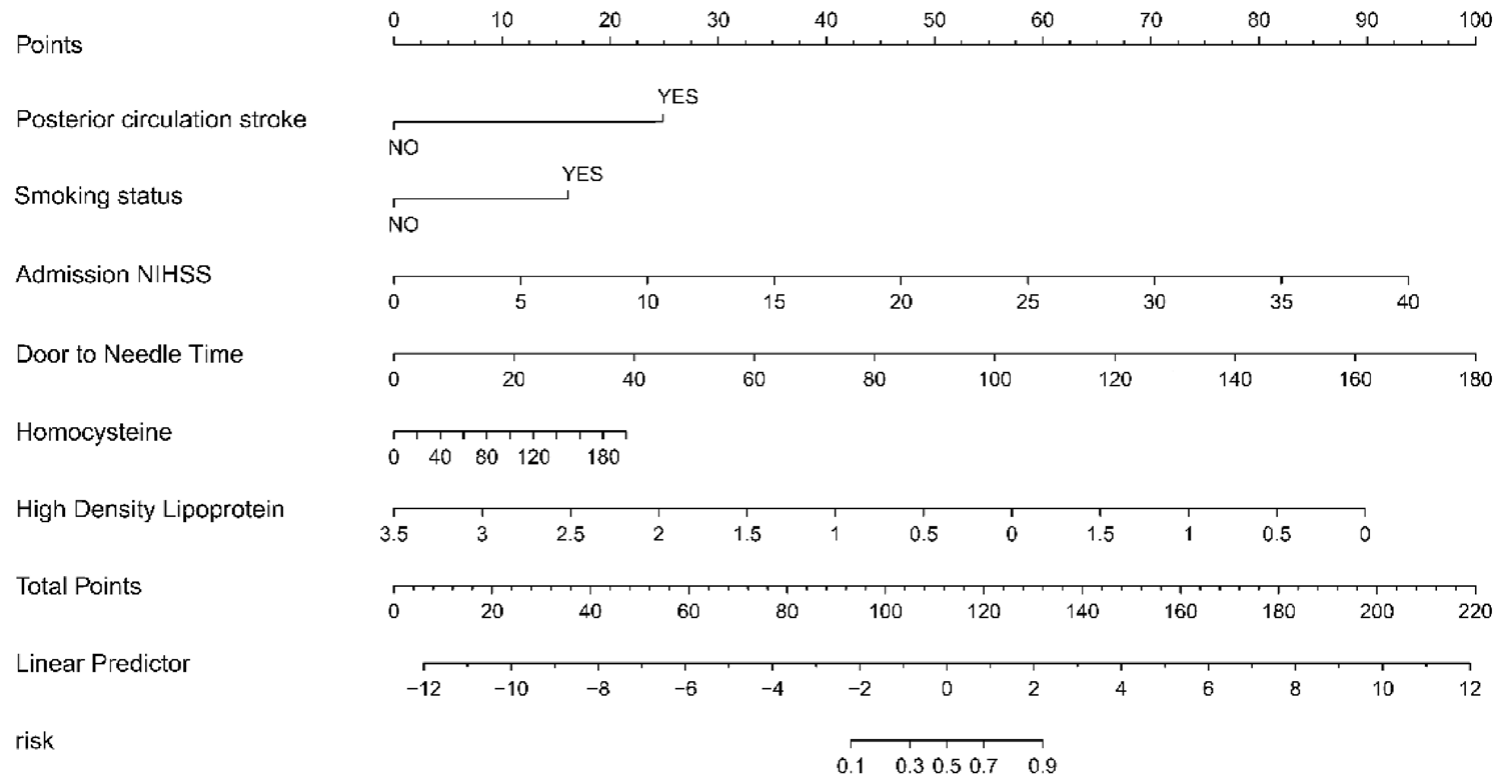
Predictive Nomogram for Thrombolysis Outcome. Nomogram developed from the multivariable logistic regression model showing weighted contributions of variables to predict ineffectiveness of thrombolytic therapy. Points are assigned for posterior circulation stroke, smoking status, admission NIHSS score, door to needle time, homocysteine, and high-density lipoprotein (HDL) levels, with total points corresponding to the probability of treatment ineffectiveness.

In this investigation, we have thoroughly constructed a nomogram, which is delineated in Fig. 3, to provide clinicians with a prognostic tool for assessing the probability of ineffectiveness following intravenous thrombolysis (IVT). The nomogram incorporates a sextet of variables established as significant through our rigorous multivariable logistic regression analysis: Posterior circulation stroke, Smoking status, Admission NIHSS score, Homocysteine level, Door to Needle Time, and High-Density Lipoprotein (HDL) concentration. Each predictor variable contributes incrementally to the total risk score, which is designed to translate directly into the estimated probability of a patient's thrombolysis response being classified as ineffective. This instrument was conceptualized to offer a quantitative approach to patient risk stratification, facilitating an enhanced individualized therapeutic strategy.

### Validation of the predictive nomogram

**Figure 4:**
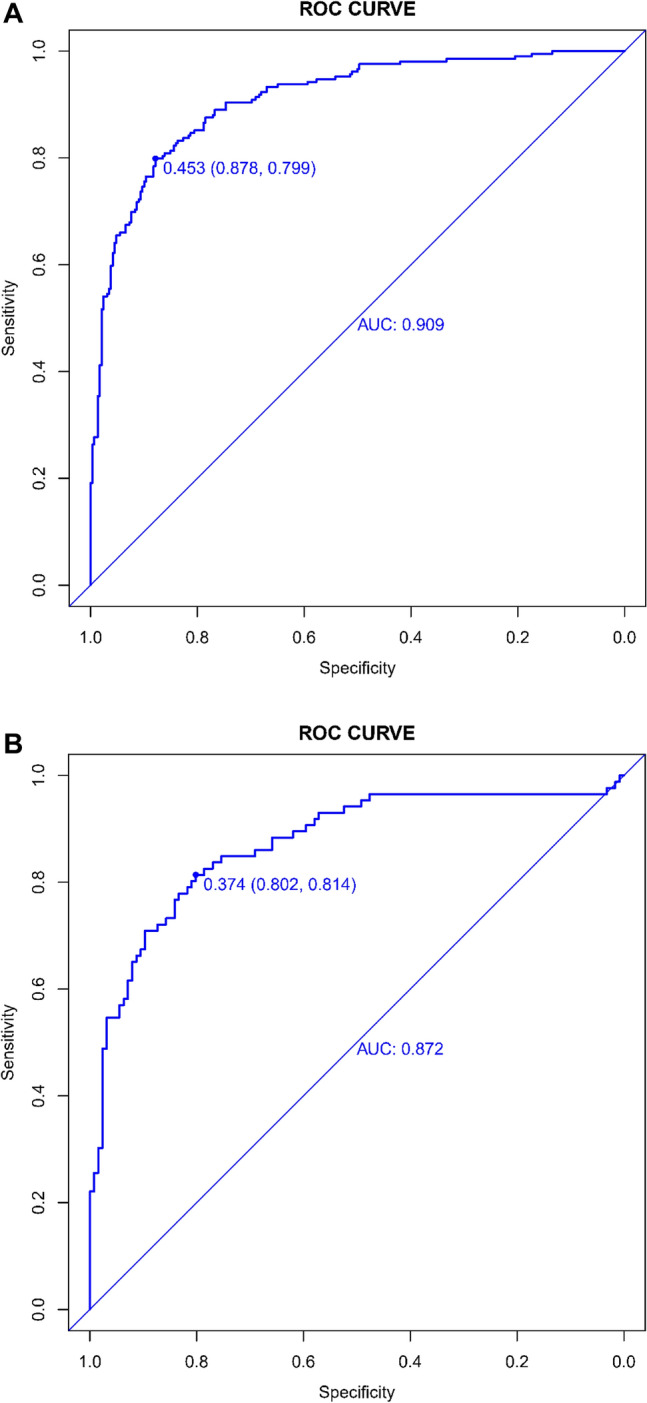
(**A**) Receiver Operating Characteristic (ROC) Curve for the Training Set. This ROC curve depicts the model's discriminative performance in the training dataset with an AUC of 0.909, showcasing its effectiveness in distinguishing between effective and ineffective thrombolysis outcomes. (**B**) Receiver Operating Characteristic (ROC) Curve for the Validation Set. The ROC curve represents the sensitivity and specificity of the ASTN-RPM in the validation set. The area under the curve (AUC) of 0.872 indicates the model's discriminative ability, with the diagonal line representing a no-discrimination scenario.

The validity and reliability of the nomogram devised to forecast the ineffectiveness of thrombolytic therapy post-intravenous treatment were subjected to rigorous verification. The nomogram's performance was quantified using the Area Under the Receiver Operating Characteristic (AUC-ROC) curve analysis. The nomogram achieved an admirable AUC of 0.909 (95% Confidence Interval [CI]: 0.883–0.935) in the training set, suggesting high discriminative capacity, as shown in Fig. 4A. Validation within an independent dataset affirmed the model's robustness, with an AUC of 0.872 (95% CI 0.821–0.924), as depicted in Fig. 4B.

**Figure 5:**
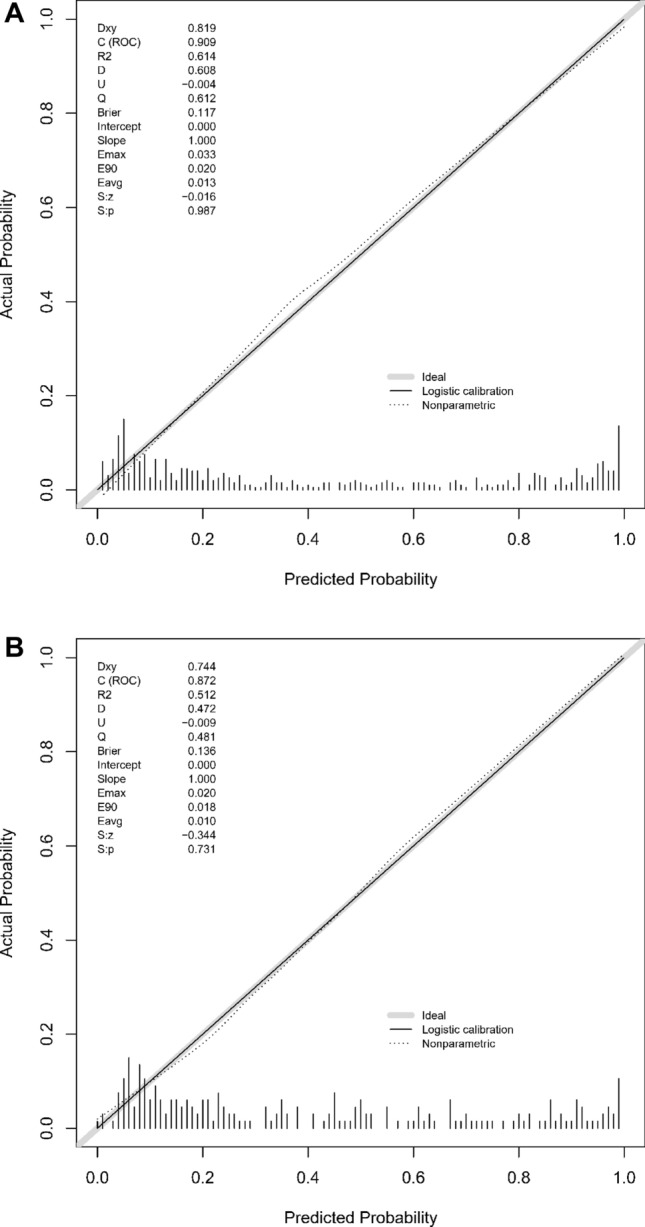
(**A**) Calibration Plot for the Training Set. Illustrates the calibration of predicted versus actual probabilities of ineffectiveness of thrombolytic therapy in the training set. The calibration line (black), nonparametric fit (dotted line), and perfect prediction line (gray) are shown, alongside a histogram of the model's predicted probabilities. (**B**) Calibration Plot for the Validation Set. Calibration plot displaying the agreement between predicted probabilities of thrombolytic therapy ineffectiveness and actual outcomes in the validation set. The plot includes a logistic calibration line, a nonparametric fit (dotted line), and the ideal reference line (gray line), with a histogram of predicted probabilities.

**Figure 6:**
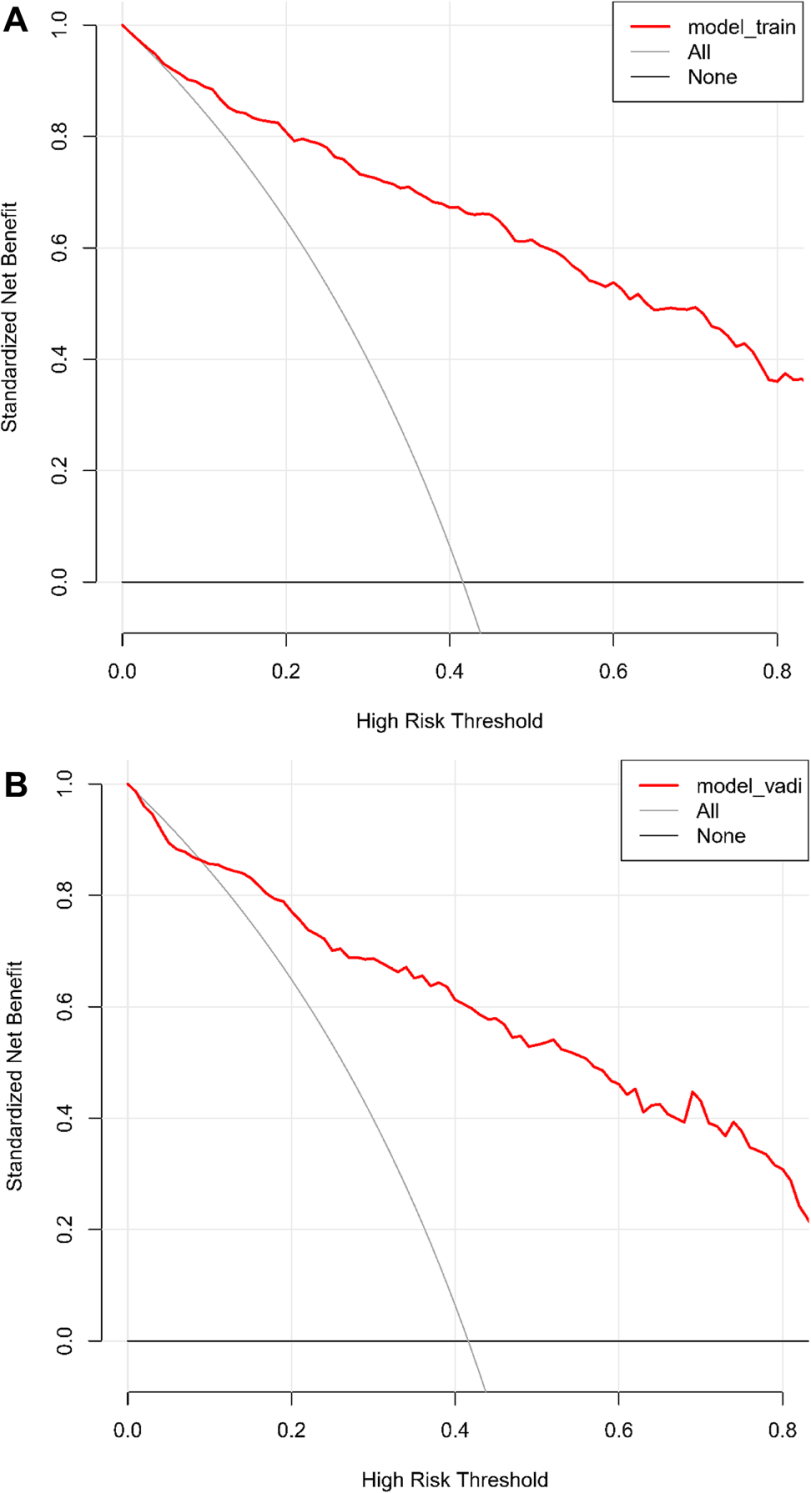
(**A**) Decision Curve Analysis for the Training Set. Decision curve analysis (DCA) for the ASTN-RPM applied to the training set. The red line illustrates the net benefit compared to the treat-all (gray line) or treat-none (black line) strategies across various thresholds. (**B**) Decision Curve Analysis for the Validation Set. Decision curve analysis (DCA) showing the net benefit of using the ASTN-RPM in the validation set across different threshold probabilities. The model's performance is shown in red, compared to the default strategies of treating all patients (gray line) or no patients (black line).

Further evaluation via calibration curves indicated a commendable concordance between the predicted probabilities and observed outcomes in both the training (Fig. 5A) and validation (Fig. 5B) sets, reflecting the nomogram's precise predictive accuracy. Decision Curve Analysis (DCA), illustrated in Fig. 6A and B, revealed a considerable net benefit across a range of practical decision thresholds, emphasizing the nomogram's clinical applicability.

The application of the nomogram in clinical scenarios demonstrated a sensitivity of 0.878 and a specificity of 0.799 in the training set, indicating the model's adeptness at identifying patients at high risk for ineffective thrombolysis (Fig. 4A). The validation set mirrored this reliability with a sensitivity of 0.802 and a specificity of 0.814 (Fig. 4B), ensuring the model's applicability across different patient cohorts. The aggregate of these analyses substantiates the nomogram's potential as a valuable tool in the clinical environment, facilitating refined risk stratification and enhancing the management of patients susceptible to an ineffective response to thrombolytic treatment.

## Discussion

Our research represents a significant contribution to the field of acute ischemic stroke treatment by presenting the ASTN-RPM, a novel predictive model for identifying patients at risk for non-responsive outcomes to thrombolytic therapy. The model's integration of both traditional risk factors and novel biochemical markers, such as Homocysteine levels, provides a multifaceted approach to risk stratification that surpasses the prognostic value offered by current clinical practices.

In comparison with existing literature, our model shares similarities in recognizing the importance of factors such as NIHSS scores and patient age, as evidenced by the IER-START nomogram by Cappellari et al.^15^, and the emphasis on onset-to-treatment time mirrored in the START model by the same authors^16^. However, the ASTN-RPM innovates by honing in on the underexplored temporal window of the first week post-therapy, a period scarcely addressed in prior studies. Moreover, our approach differs from the artificial neural network-based models utilized by Chung et al.^17^ and the traditional regression methods adopted by Deng et al.^18^. Our application of LASSO regression caters to the high-dimensional nature of the dataset while mitigating the risks of model complexity and overfitting, whereas the aforementioned studies inclined towards more complex machine learning algorithms.

Unique to our research is the consideration of biomarkers such as homocysteine levels and high-density lipoprotein, which have not been traditionally factored into thrombolysis effectiveness prediction models. While Kim et al.^19^ developed the S-SMART score focusing on clinical features and treatment types, our model expands the predictive scope to biochemical domains. Despite our model's robust AUC values within our training and validation cohorts, we acknowledge, as do Lv et al.^20^ and Li et al.^21^, that external validation remains a critical measure of a model’s applicability across diverse populations. Therefore, ASTN-RPM requires further validation in a broader, multi-centric cohort to ensure its predictive precision and practical utility. When considering machine learning models, we also recognize that models integrating clinical and imaging data, as discussed by Ramos et al.^22^and Zhang et al.^23^, may offer enhanced predictive accuracy. While the ASTN-RPM currently does not incorporate imaging data directly, future iterations could potentially benefit from integrating such multimodal information to enrich its predictive capabilities.

A major strength of our model lies in its methodological rigor, which is evident from the high AUC values indicating excellent predictive performance. This contrasts favorably with previous studies that have reported lower discriminative abilities^18,24,25^, affirming the superior predictive nature of ASTN-RPM. The model's ability to stratify patients effectively into responders and non-responders not only holds potential for optimizing clinical decision-making but also for potentially tailoring patient-specific therapeutic approaches.

Our findings offer a fresh perspective on patient selection for thrombolysis, suggesting that a more nuanced assessment of risk factors, including those identified in our model, could lead to improved clinical outcomes. Such an approach could also foster a more judicious use of healthcare resources by identifying patients who may benefit from alternative or adjunctive therapies. In comparison with the current literature, our study expands the understanding of thrombolysis ineffectiveness by quantitatively integrating risk factors into a robust nomogram. It provides a novel, evidence-based tool for clinicians in the acute care setting and serves as a foundation for further clinical inquiry and intervention development. In summary, the ASTN-RPM stands as a testament to the potential of predictive modeling in revolutionizing stroke care. By providing a data-driven, patient-centric approach, our model encapsulates the progression towards precision medicine in stroke management, offering not just a statistical prediction but a pathway to enhanced patient care and outcomes.

While the ASTN-RPM demonstrates promise, its retrospective design inherently limits the ability to establish causality. This limitation is significant in understanding the mechanistic underpinnings of thrombolysis response. Moreover, retrospective studies may encounter biases such as selection bias and information bias, which could affect the interpretation of results. To mitigate these limitations, prospective studies are needed to confirm our findings and potentially illuminate causative relationships. External validation is another pivotal step towards broad application. Our cohort, derived from a single center, might not fully represent the global population. Multi-center studies spanning diverse demographics are imperative to ascertain the model's performance across different ethnicities and healthcare settings, ensuring its utility is not confined to a homogenous group.

Our model did not incorporate emerging biomarkers, which have shown potential in other studies. Inclusion of novel molecular markers or imaging findings in future models could significantly bolster their prognostic accuracy. The burgeoning field of genomics presents another horizon for research. Genetic factors may profoundly influence thrombolysis outcomes, and incorporating genetic profiling into the ASTN-RPM could provide an even more nuanced prediction. The exploration of pharmacogenomics may yield insights into personalized thrombolytic therapy, potentially reducing the prevalence of non-responders. Additionally, as stroke management evolves with technological advancements like mechanical thrombectomy, our model must be dynamic, adapting to new treatment paradigms. It may be pertinent to assess how such treatments interact with the variables in our model and recalibrate accordingly. Longitudinal studies could further delineate the long-term outcomes of patients identified as non-responders by our model, offering a clearer picture of the model’s impact on the trajectory of stroke recovery and rehabilitation.

In conclusion, the ASTN-RPM encapsulates our commitment to advancing stroke care through precision medicine. By identifying patients unlikely to benefit from traditional thrombolytic therapy, we pave the way for more personalized and effective treatment strategies. This model is a testament to the potential of leveraging comprehensive clinical data, pioneering a new direction in the management of acute ischemic stroke.

### Supplementary Information

Supplementary Table 1.

## Data Availability

The data generated and analyzed in this study are not open to the public because of privacy and ethical considerations. However, they can be obtained from the corresponding author upon a reasonable request. The article encompasses all pertinent data underpinning the results of this research.
