# Supplementary material for: Predicting ineffective thrombolysis in acute ischemic stroke with clinical and biochemical markers
Source: Sci Rep. 2024 Jun 11;14:13424. doi: 10.1038/s41598-024-64413-w (PMC11166982; doi:10.1038/s41598-024-64413-w)
Supplement: Supplementary file 1 — Supplementary Table 1. [file 41598_2024_64413_MOESM1_ESM.docx]

Supplemental Table 1. Comparison of baseline characteristics between the training and validation sets.

| Variables | Total  (n = 709) | Training set  (n = 497) | validation set  (n = 212) | p |
| --- | --- | --- | --- | --- |
| Prognosis, n (%) | |  |  | 0.776 |
| Thrombolysis effective | 414 (58) | 288 (58) | 126 (59) |  |
| Thrombolysis ineffective | 295 (42) | 209 (42) | 86 (41) |  |
| TOAST classification of stroke etiology, n (%) | |  |  | 0.741 |
| Large artery atherosclerosis | 536 (76) | 371 (75) | 165 (78) |  |
| Small vessel disease | 108 (15) | 75 (15) | 33 (16) |  |
| Cardioembolic | 55 (8) | 43 (9) | 12 (6) |  |
| Other determined etiology | 5 (1) | 4 (1) | 1 (0) |  |
| Undetermined etiology | 5 (1) | 4 (1) | 1 (0) |  |
| Posterior circulation stroke, n (%) | |  |  | 0.256 |
| NO | 543 (77) | 387 (78) | 156 (74) |  |
| YES | 166 (23) | 110 (22) | 56 (26) |  |
| Smoking status, n (%) | |  |  | 0.464 |
| NO | 368 (52) | 253 (51) | 115 (54) |  |
| YES | 341 (48) | 244 (49) | 97 (46) |  |
| Drinking status, n (%) | |  |  | 0.864 |
| NO | 480 (68) | 335 (67) | 145 (68) |  |
| YES | 229 (32) | 162 (33) | 67 (32) |  |
| History of hypertension, n (%) | |  |  | 0.817 |
| NO | 268 (38) | 186 (37) | 82 (39) |  |
| YES | 441 (62) | 311 (63) | 130 (61) |  |
| Gender, n (%) | |  |  | 0.949 |
| Female | 237 (33) | 167 (34) | 70 (33) |  |
| male | 472 (67) | 330 (66) | 142 (67) |  |
| Admission impaired consciousness, n (%) | | |  | 0.656 |
| NO | 574 (81) | 405 (81) | 169 (80) |  |
| YES | 135 (19) | 92 (19) | 43 (20) |  |
| Prior stroke history, n (%) | | |  | 0.695 |
| NO | 619 (87) | 436 (88) | 183 (86) |  |
| YES | 90 (13) | 61 (12) | 29 (14) |  |
| Diabetes mellitus, n (%) | |  |  | 0.049 |
| NO | 547 (77) | 394 (79) | 153 (72) |  |
| YES | 162 (23) | 103 (21) | 59 (28) |  |
| Atrial fibrillation history, n (%) | | |  | 0.76 |
| NO | 623 (88) | 435 (88) | 188 (89) |  |
| YES | 86 (12) | 62 (12) | 24 (11) |  |
| Coronary artery disease history, n (%) | |  |  | 0.272 |
| NO | 552 (78) | 393 (79) | 159 (75) |  |
| YES | 157 (22) | 104 (21) | 53 (25) |  |
| History of hyperlipidemia, n (%) | | |  | 0.432 |
| NO | 702 (99) | 493 (99) | 209 (99) |  |
| YES | 7 (1) | 4 (1) | 3 (1) |  |
| Admission NIHSS, Median (Q1,Q3) | 3 (1, 9) | 3 (1, 10) | 3 (1, 8.25) | 0.272 |
| Age (years), Median (Q1,Q3) | 65 (55, 71) | 65 (55, 71) | 65 (55, 71) | 0.899 |
| Height (cm), Median (Q1,Q3) | 170 (162, 173) | 170 (162, 173) | 170 (162, 173) | 0.855 |
| Weight (Kg), Median (Q1,Q3) | 70 (60, 78) | 70 (61, 78) | 70 (60, 78) | 0.198 |
| Body Mass Index, Median (Q1,Q3) | 25.06 (22.49, 27.17) | 25.18 (22.6, 27.12) | 24.58 (21.8, 27.24) | 0.21 |
| Door to Needle Time (min), Median (Q1,Q3) | 49 (35, 73) | 49(35, 73) | 49 (35, 71) | 0.724 |
| Systolic blood pressure (mmHg), Median (Q1,Q3) | 146 (134, 160) | 145 (134, 157) | 150 (135, 162) | 0.008 |
| Diastolic blood pressure (mmHg), Median (Q1,Q3) | 82 (76, 89) | 82 (76, 89) | 83 (77, 90) | 0.208 |
| White blood cell count (×10^9^/L), Median (Q1,Q3) | 7.4 (6.01, 8.93) | 7.31 (5.96, 8.75) | 7.53 (6.09, 9.27) | 0.211 |
| Hemoglobin (g/L), Mean ±SD | 144.19 ± 17.41 | 144.06 ± 17.57 | 144.48 ± 17.06 | 0.766 |
| Platelet count (×10^9^/L), Median (Q1,Q3) | 216 (179, 256) | 213 (175, 255) | 224 (185, 259) | 0.141 |
| Neutrophil count (×10^9^/L), Median (Q1,Q3) | 4.49 (3.44, 5.84) | 4.49 (3.46, 5.86) | 4.5 (3.4, 5.82) | 0.771 |
| Monocyte count (×10^9^/L), Median (Q1,Q3) | 0.52 (0.4, 0.64) | 0.52 (0.4, 0.64) | 0.52 (0.41, 0.63) | 0.575 |
| Platelet to Neutrophil Ratio, Median (Q1,Q3) | 47.55 (35.61, 65.31) | 47.37 (35.61, 65.52) | 49.95 (36.06, 64.45) | 0.968 |
| Neutrophil percentage, Median (Q1,Q3) | 62.9 (53.8, 72.8) | 64 (54.3, 73.1) | 62.1 (52.98, 71.95) | 0.187 |
| Lymphocyte percentage, Median (Q1,Q3) | 1.87 (1.36, 2.5) | 1.83 (1.35, 2.43) | 2 (1.41, 2.71) | 0.065 |
| Neutrophil to Lymphocyte Ratio, Median (Q1,Q3) | 2.25 (1.5, 3.82) | 2.28 (1.54, 3.85) | 2.13 (1.42, 3.66) | 0.283 |
| Systemic Immune Inflammation Index, Median (Q1,Q3) | 493.14 (321.7, 819.31) | 490.2 (323.3, 823.39) | 499.3 (316.19, 795.87) | 0.543 |
| Prothrombin Time (s), Median (Q1,Q3) | 10.8 (10.3, 11.5) | 10.8 (10.3, 11.5) | 10.85 (10.2, 11.4) | 0.328 |
| Prothrombin Activity (%), Median (Q1,Q3) | 100.7 (93.9, 108.7) | 100.4 (93.3, 108.7) | 102 (95.85, 108.78) | 0.071 |
| Activated Partial Thromboplastin Time (s), Median (Q1,Q3) | 26.4 (24.6, 29) | 26.4 (24.6, 29) | 26.5 (24.6, 29.02) | 0.75 |
| Fasting Blood Glucose (mmol/L), Median (Q1,Q3) | 6.86 (5.81, 8.97) | 6.82 (5.79, 8.85) | 7 (5.86, 9.2) | 0.489 |
| Brain natriuretic peptide (pg/ml), Median (Q1,Q3) | 28.3 (9.8, 114) | 29 (9.7, 121) | 25.2 (10.65, 91.97) | 0.407 |
| Lactate Dehydrogenase (U/L), Median (Q1,Q3) | 399 (233.9, 490.61) | 403.75 (239, 492.82) | 391.88 (226, 487.36) | 0.499 |
| Creatinine (μmol/L), Median (Q1,Q3) | 67.1 (57.44, 78.73) | 66.8 (56.84, 79.1) | 67.42 (58.99, 78.45) | 0.462 |
| Uric Acid (μmol/L), Median (Q1,Q3) | 323.58 (264.9, 395.47) | 324.2 (266.12, 399.4) | 322.85 (263.08, 387.18) | 0.298 |
| Total Cholesterol (mmol/L), Median (Q1,Q3) | 4.56 (3.92, 5.3) | 4.56 (3.94, 5.31) | 4.58 (3.88, 5.24) | 0.979 |
| Triglycerides (mmol/L), Median (Q1,Q3) | 1.28 (0.92, 1.8) | 1.32 (0.94, 1.81) | 1.24 (0.9, 1.74) | 0.125 |
| Low-Density Lipoprotein (mmol/L), Median (Q1,Q3) | 2.7 (2.12, 3.28) | 2.71 (2.11, 3.28) | 2.67 (2.13, 3.25) | 0.79 |
| HDL/LDL ratio, Median (Q1,Q3) | 0.37 (0.29, 0.48) | 0.36 (0.28, 0.47) | 0.38 (0.31, 0.5) | 0.094 |
| Homocysteine (μmol/L), Median (Q1,Q3) | 14.86 (11.5, 25.2) | 14.64 (11.38, 24.83) | 15.2 (11.88, 25.53) | 0.566 |
| High Density Lipoprotein (mmol/L), Median (Q1,Q3) | 1.02 (0.79, 1.23) | 1 (0.75, 1.22) | 1.04 (0.86, 1.23) | 0.108 |
| Total Cholesterol/HDL Cholesterol ratio, Median (Q1,Q3) | 4.47 (3.65, 5.5) | 4.55 (3.68, 5.64) | 4.38 (3.58, 5.19) | 0.091 |
